# Uncertainty Relations in the Madelung Picture

**DOI:** 10.3390/e24010020

**Published:** 2021-12-23

**Authors:** Moise Bonilla-Licea, Dieter Schuch

**Affiliations:** 1 Departamento de Física y Matemáticas, Universidad Iberoamericana, Prolongación Paseo de la Reforma 880, Mexico City 01219, Mexico; 2 Institute of Theoretical Physics, Goethe-University Frankfurt, Max-von-Laue-Str. 1, D-60438 Frankfurt am Main, Germany; schuch@em.uni-frankfurt.de

**Keywords:** Madelung formalism, complex quantum hydrodynamics, coherent states

## Abstract

Madelung showed how the complex Schrödinger equation can be rewritten in terms of two real equations, one for the phase and one for the amplitude of the complex wave function, where both equations are not independent of each other, but coupled. Although these equations formally look like classical hydrodynamic equations, they contain all the information about the quantum system. Concerning the quantum mechanical uncertainties of position and momentum, however, this is not so obvious at first sight. We show how these uncertainties are related to the phase and amplitude of the wave function in position and momentum space and, particularly, that the contribution from the phase essentially depends on the position–momentum correlations. This will be illustrated explicitly using generalized coherent states as examples.

## 1. Introduction

Following numerous attempts at the beginning of the 20th century, a satisfactory solution to the wave–particle duality problem was found by Heisenberg, Born, and Jordan [1,2,3] in 1925 with matrix mechanics and, almost simultaneously, by Schrödinger [4,5,6] with wave mechanics. At a glance, both theories look quite different, but their physical equivalence was shown already in the very beginning [7]. Both approaches also have in common the use of complex quantities and the explicit occurrence of $i=\sqrt{-1}$. As C.N. Yang emphasized in his talk on the occasion of Schrödinger’s 100th anniversary [8]: “...With matrix mechanics and wave mechanics, however, the situation dramatically changed. Complex numbers became a conceptual element of the very foundations of physics: the fundamental equations of matrix mechanics and wave mechanics:

(1) $$\begin{array}{cc} pq-qp & =-i\hbar \end{array}$$

(2) $$\begin{array}{cc} i\hbar \frac{\partial \psi }{\partial t} & =H\psi \end{array}$$

both explicitly contain the imaginary unit $i=\sqrt{-1}$”.

Considering, particularly, Schrödinger’s wave mechanics, the corresponding wave equation is linear, so one could assume that changing from a real to a complex wave function would not cause much difference; it would just produce two (almost, apart from the real potential *V*) identical equations for real and imaginary parts of the complex wave function. However, this is not the case, because $i=\sqrt{-1}$ occurs explicitly in the Schrödinger equation, i.e., in position space. In the following, we particularly consider, in detail, the one-dimensional case; the extension to higher dimensions is straightforward.

(3) $$i\hbar \frac{\partial }{\partial t}\psi \left(x,t\right)=-\frac{\hbar^{2}}{2m}\frac{\partial^{2}}{\partial x^{2}}\psi \left(x,t\right)+V\psi \left(x,t\right).$$

Consequently, the time-derivative of the imaginary part of ψ corresponds to the second spatial derivative of the real part of ψ, and vice versa (see also [9,10]), i.e., real and imaginary parts are not independent of each other, but coupled.

This coupling is even more obvious in a formulation of wave mechanics that was introduced by Madelung [11] shortly after Schrödinger’s communications. He considered a polar form of the complex wave function, i.e.,

(4) $$\psi \left(x,t\right)=\sqrt{\rho_{x}\left(x,t\right)}\exp\left[\frac{i}{\hbar }S_{x}\left(x,t\right)\right]$$

with the amplitude $\sqrt{\rho_{x}}$, where $\rho_{x}=\psi^{*}\left(x,t\right)\psi \left(x,t\right)$, and the phase $\frac{S_{x}\left(x,t\right)}{\hbar }$. Inserting this form into the time-dependent Schrödinger Equation (3) provides two (real) equations, one for the phase $S_{x}\left(x,t\right)$,

(5) $$\frac{\partial S_{x}}{\partial t}+\frac{1}{2m}{\left(\frac{\partial S_{x}}{\partial x}\right)}^{2}+V_{qu,x}+V=0,$$

a kind of modified Hamilton–Jacobi equation for the action $S_{x}\left(x,t\right)$, which corresponds to the real part of the Schrödinger equation, including the so-called “quantum potential” $V_{qu,x}=-\frac{\hbar^{2}}{2m}\frac{\frac{\partial^{2}\sqrt{\rho_{x}}}{\partial x^{2}}}{\sqrt{\rho_{x}}}$, and one for the amplitude or density $\rho_{x}$,

(6) $$\frac{\partial \rho_{x}\left(x,t\right)}{\partial t}+\frac{\partial }{\partial x}\left(\rho_{x}\upsilon_{R}\left(x,t\right)\right)=0$$

corresponding to the imaginary part of Equation (3), with the velocity field (The meaning of the subscript “*R*” becomes obvious from Equation (23) with $\upsilon_{R}=\frac{1}{m}P_{R}$.) $\upsilon_{R}=\frac{1}{m}\frac{\partial S_{x}}{\partial x}$.

Because Equation (5), for the phase, contains explicitly the density $\rho_{x}$ via the term $V_{qu}$, and Equation (6), for the density, contains the phase via $\upsilon_{R}$, the two components of the complex wave function are, again, obviously coupled.

In 1951, Mrowka showed how a generalization of Madelung’s formulation can be used as a basis for an axiomatic derivation of Schrödinger’s equation, including a magnetic field and an extension to the nonrelativistic Pauli equation [12], as well as a generalization to include the relativistic case [13].

Shortly afterwards, in 1952, Bohm [14,15] independently took up Madelung’s idea, but tried to give it a deterministic interpretation, assuming that the integration of Madelung’s velocity $\upsilon_{R}$ would provide trajectories that are real paths followed by physical particles. (For a comprehensive review of the criticism of Bohm’s interpretation see [16,17,18,19] and the references cited therein; a consistent explanation of the Bohmian trajectories in probabilistic terms was given recently in [20].) Despite the ontological problems, the application of the Bohmian approach developed very useful numerical methods for the treatment of molecular reactions, tunnelling, and diffraction phenomena (see [21] for a review).

Common to all the above-mentioned hydrodynamic formulations is their exclusive restriction to configuration space, a point Pauli and Heisenberg criticised with respect to Bohm’s approach [16,22]. To achieve a complete dynamical picture, it must be possible to formulate the hydrodynamic equations in any representation. Recently, Bonilla and Schuch clarified how Madelung’s hydrodynamic picture can be extended consistently to include both position and momentum representations. In this context, the connection of the position and momentum uncertainties, and the corresponding uncertainty product with the hydrodynamic quantities in the Madelung picture, need further examination, particularly regarding what the contributions of phase and amplitude to these quantities are. This will be analysed in detail for the position as well as for the momentum space in this paper.

For this purpose, in Section 2, a brief description of the Madelung picture and its relation to the Heisenberg and Schrödinger picture is given. Particularly, the explicit expressions for the complex Madelung quantities in position and momentum space that are used in the following are provided. In Section 3, it is shown how, in position space, the momentum uncertainty has particular contributions from the phase and amplitude of the wave function and that, vice versa, a corresponding relation exists in momentum space for the position uncertainty. The connection with the correlation of position and momentum uncertainties will also be discussed. These findings will be illustrated through an example in Section 4, by considering Gaussian wave packet solutions of the time-dependent Schrödinger equation. Section 5 closes our discussion by summarising the results.

## 2. Madelung Picture

Let us briefly outline the main characteristics of the Madelung picture. For instance, in the Heisenberg picture, the observables *F* are time-dependent, ${\left(F\right)}_{H}={\hat{F}}_{H}\left(t\right)$, while the quantum state is time-independent, $|\psi_{0}\rangle$. In the Schrödinger picture, the opposite holds, i.e., the observable is time-independent, ${\left(F\right)}_{S}={\hat{F}}_{S}$, whereas the state is time-dependent, |ψ(t)〉. Regarding the Madelung picture, **we assume the observables are mapped onto time-dependent complex functions** (for more details, see also [19]), ${\left(F\right)}_{M}=F\left(a,t\right)=F_{R}\left(a,t\right)+iF_{I}\left(a,t\right)$, where *a* denotes, e.g., position or momentum. Hereafter, position and momentum representations will be exclusively considered, since the purpose is to discuss the basic relations of quantum mechanics from the point of view of the Madelung formulation. However, the underlying method of our **Madelung picture can be applied to any kind of representation** (for details, see [19]). **We consider, explicitly, the probability density $\rho_{a}=\rho_{a}\left(a,t\right)$ as well**.

All three pictures provide the same mean values that are related via

(7) 〈F〉(t)=〈ψ0|F^H(t)|ψ0〉=〈ψ(t)|F^S|ψ(t)〉=∫−∞+∞daaρa(a,t)FM(a,t).

where the positive definite density $\rho_{a}\left(a,t\right)$ is given by

(8) $$\rho_{a}\left(a,t\right)=\langle \psi_{0}|a\left(t\right)\rangle \langle a\left(t\right)|\psi_{0}\rangle =\langle \psi \left(t\right)|a\rangle \langle a|\psi \left(t\right)\rangle$$

and the Madelung quantities ${\left(F\right)}_{M}=F\left(a,t\right)$ are obtained via

(9) $$F\left(a,t\right)=\frac{\langle a\left(t\right)|{\hat{F}}_{H}\left(t\right)|\psi_{0}\rangle }{\langle a\left(t\right)|\psi_{0}\rangle }=\frac{\langle a|{\hat{F}}_{S}|\psi \left(t\right)\rangle }{\langle a|\psi (t)\rangle }.$$

Since the mean values of observables must be real, according to (7), the mean value of the imaginary part of the Madelung quantities must vanish:

(10) ∫−∞+∞daaρa(a,t)FI(a,t)=0.

This might raise the question, what is the purpose of considering the imaginary part if the mean value seems to not be affected by it? The answer to this question will become apparent in Section 3.

It should also be noted that the commutation relations are preserved, which implies, in the Madelung picture, that in general, the quantities ${\left(XP\right)}_{M}$ and ${\left(PX\right)}_{M}$ are not simply the product of the two complex quantities ${\left(X\right)}_{M}$${\left(P\right)}_{M}$. Particularly for X, representing position and P momentum, the products obey the commutation relation according to

(11) 〈X^H(t),P^H(t)−〉=〈X^S,P^S−〉=∫−∞+∞daaρa(a,t)(XP)M−(PX)M=iℏ

where ${\left[\cdot ,\cdot \right]}_{-}$ denotes the commutator.

As the commutation relations hold, also in the Madelung picture, the time-evolution of the mean values obeys the classical equations of motion according to the Ehrenfest theorem.

### 2.1. Position and Momentum Spaces

We consider a quantum system with mass *m* obeying a quadratic Hamiltonian of the form $\hat{H}=\hat{T}+V\left(\hat{X}\right)=\frac{1}{2m}{\hat{P}}^{2}+\frac{1}{2}m\omega^{2}{\hat{X}}^{2}$ in the position and momentum representations. It is worth noticing that constant terms and terms that are linear in position **can be included without problem** into the potential, but are not relevant to the following discussion. Furthermore, for a **general potential**, it can be expressed as a power series of the position variable and treated iteratively.

As Madelung obtained his equations by separating the Schrödinger equation into two equations (one for phase and one for amplitude of the time-dependent wave function), we also base our discussion on the Schrödinger picture, considering now, however, the complex quantities defined above.

In the Schrödinger picture, the operators representing the relevant observables $\hat{X}$, $\hat{P}$, $\hat{V}$, $\hat{T}$, and $\hat{H}$ are given by:

Position space

(12) $$\begin{array}{ccc} \langle x|\hat{X} & = & x\in R, \end{array}$$

(13) $$\begin{array}{ccc} \langle x|\hat{P} & = & -i\hbar \frac{\partial }{\partial x}, \end{array}$$

(14) $$\begin{array}{ccc} \langle x|V\left(\hat{X}\right) & = & V(x), \end{array}$$

(15) $$\begin{array}{ccc} \langle x|\hat{T} & = & -\frac{\hbar^{2}}{2m}\frac{\partial^{2}}{\partial x^{2}}, \end{array}$$

(16) $$\begin{array}{ccc} \langle x|\hat{H} & = & i\hbar \frac{\partial }{\partial t}. \end{array}$$

Momentum space

(17) $$\begin{array}{ccc} \langle p|\hat{X} & = & i\hbar \frac{\partial }{\partial p}, \end{array}$$

(18) $$\begin{array}{ccc} \langle p|\hat{P} & = & p\in R, \end{array}$$

(19) $$\begin{array}{ccc} \langle p|V\left(\hat{X}\right) & = & V(i\hbar \frac{\partial }{\partial p}), \end{array}$$

(20) $$\begin{array}{ccc} \langle p|\hat{T} & = & \frac{p^{2}}{2m}, \end{array}$$

(21) $$\begin{array}{ccc} \langle p|\hat{H} & = & i\hbar \frac{\partial }{\partial t}. \end{array}$$

In their respective representations, position $\hat{X}$, and momentum $\hat{P}$, as well as observables that explicitly only depend on these variables, correspond to real quantities, whereas all other quantities correspond to differential operators (which can also be imaginary).

Regarding the Madelung picture, the quantum state is hereafter expressed using a polar ansatz according to $\langle a|\psi \left(t\right)\rangle =\sqrt{\rho_{a}\left(a,t\right)}\exp\left[\frac{i}{\hbar }S_{a}\left(a,t\right)\right]$, where *a* denotes the position or momentum-representation variables *x* or *p*.

According to definition (9), the explicit expressions for $\hat{X}$, $\hat{P}$, $\hat{V}$, $\hat{T}$, and $\hat{H}$ are now given by the quantities:

Position space

(22) $$\begin{array}{ccc} X & = & x\in R, \end{array}$$

(23) $$\begin{array}{ccc} P & = & \frac{\partial S}{\partial x}-i\frac{\hbar }{2}\frac{\frac{\partial \rho_{x}}{\partial x}}{\rho_{x}}=P_{R}+iP_{I}, \end{array}$$

(24) $$\begin{array}{ccc} V & = & \frac{m\omega^{2}}{2}x^{2}, \end{array}$$

(25) $$\begin{array}{ccc} T & = & \frac{1}{2m}\left(P^{2}-i\hbar \frac{\partial P}{\partial x}\right), \end{array}$$

(26) $$\begin{array}{ccc} H & = & -\frac{\partial S}{\partial t}+i\frac{\hbar }{2}\frac{\frac{\partial \rho_{x}}{\partial t}}{\rho_{x}}. \end{array}$$

Momentum space

(27) $$\begin{array}{ccc} \;X & = & -\frac{\partial S}{\partial p}+i\frac{\hbar }{2}\frac{\frac{\partial \rho_{p}}{\partial p}}{\rho_{p}}=X_{R}+iX_{I}, \end{array}$$

(28) $$\begin{array}{ccc} P & = & p\in R, \end{array}$$

(29) $$\begin{array}{ccc} V & = & \frac{m\omega^{2}}{2}\left(X^{2}+i\hbar \frac{\partial X}{\partial p}\right), \end{array}$$

(30) $$\begin{array}{ccc} T & = & \frac{p^{2}}{2m}, \end{array}$$

(31) $$\begin{array}{ccc} H & = & -\frac{\partial S}{\partial t}+i\frac{\hbar }{2}\frac{\frac{\partial \rho_{p}}{\partial t}}{\rho_{p}}. \end{array}$$

As in the Schrödinger picture, in the Madelung picture, position and momentum and quantities depending only on these variables correspond to real quantities in their respective representations, whereas all other quantities are now complex.

It is important to point out that, in the Madelung picture, in general, the square of an observable does not correspond to the square of the corresponding complex quantity, i.e., ${\left(F^{2}\right)}_{M}\neq {\left({\left(F\right)}_{M}\right)}^{2}$. This applies particularly to the kinetic energy in position representations, as $\frac{\langle x|{\hat{P}}^{2}|\psi \left(t\right)\rangle }{\langle x|\psi (t)\rangle }={\left(\frac{\langle x|\hat{P}|\psi \left(t\right)\rangle }{\langle x|\psi (t)\rangle }\right)}^{2}-i\hbar \frac{\partial }{\partial x}\left(\frac{\langle x|\hat{P}|\psi \left(t\right)\rangle }{\langle x|\psi (t)\rangle }\right)$, or, according to definition (9), ${\left(P^{2}\right)}_{M}=P^{2}-i\hbar \frac{\partial P}{\partial x}\neq P^{2}$. A similar argument applies to ${\left(X^{2}\right)}_{M}$ in momentum space. The occurrence of the additional (complex) derivative-term is due to the non-locality of quantum mechanics (for a more detailed discussion, see also [19]).

The Madelung quantities (9) formally resemble the definition of the weak values [23,24,25]. Nevertheless, it is important to point out that although both the weak values formulation and the Madelung picture make use of similar mathematical expressions, they are not the same. Indeed, the essence of a theory is not reduced merely to a collection of mathematical objects and equations, but the ontology also plays an important role, e.g., in the way these objects and equations have to be understood and interpreted. In this sense, even though our Madelung quantities match the structure of the weak values [26], the ontology is totally different. Indeed, in the Madelung picture, contrary to the weak value formulation, there is no measurement process under consideration, nor are there preselected and postselected states [27]. Moreover, the Madelung picture is based on the same ontological grounds of conventional quantum mechanics as the Schrödinger and Heisenberg pictures are. The aim of the Madelung picture is not to give a more detailed description of the measurement process than the conventional quantum mechanics. Instead, the main goal is a systematic description of the quantum dynamics through the use of hydrodynamical objects for any representation; in the same way, the Schrödinger picture describes the quantum dynamics using a time-dependent state that, once projected on a given representation, presents ondulatory or wave-like properties. Likewise, the Heisenberg picture describes the quantum dynamics using time-dependent operators whose entries, once the representation is chosen, exhibit the properties of Fourier components of oscillation modes. (Note that the reason for coining the name ”Madelung picture”, and not simply using the term ”Madelung interpretation”, is that, as Bohm pointed out in [14], Madelung did not push forward the logical rationale of his interpretation).

It is important to point out that a distinct feature of the dynamics of our Madelung quantities is that they obey a transport equation in both position and momentum representation; for a detailed discussion, we refer to [28]. This is particularly helpful and important when dealing with potentials with discontinuities, as in tunnelling problems. It should also be noted that the character of the transport equations holds, even for a general potential.

## 3. Quantum Fluctuations in Position and Momentum Space

First of all, let us consider a given observable $\hat{F}$, and let us compute its uncertainty by means of its associated Madelung quantity F (see Equation (9)). It is clear that, for the space (a,t),

(32) $$\sigma_{F}^{2}=\langle \psi |{\hat{F}}^{2}|\psi \rangle -{\langle F\rangle }^{2}=\int_{-\infty }^{+\infty }\rho_{a}{\left(F^{2}\right)}_{M}da-{\langle F\rangle }^{2}.$$

Moreover, recall that the average of the imaginary part of a Madelung quantity cancels out (see Section 2). Then, Equation (32) is valid for any observable. The particular expression of the uncertainty will depend on the structure of the real part of the Madelung quantity associated with the square of such an observable. This, of course, depends also on the representation that is chosen, since the effect of an operator generally changes from representation to representation.

Now, regarding the momentum uncertainty $\sigma_{p}^{2}$ in the position representation in the Madelung picture, i.e.,

(33) $$\sigma_{p}^{2}=\langle \psi |{\hat{P}}^{2}|\psi \rangle -{\langle p\rangle }^{2}=\int_{-\infty }^{+\infty }\rho_{x}{\left(P^{2}\right)}_{M}dx-{\langle p\rangle }^{2}$$

where ${\left(P^{2}\right)}_{M}$ is related to the kinetic energy T via $T=\frac{1}{2m}{\left(P^{2}\right)}_{M}=T_{R}+iT_{I}$. As already mentioned in (10), the mean value of the imaginary part vanishes, $\int_{-\infty }^{+\infty }\rho_{x}T_{I}dx=0$, leaving

(34) $$\sigma_{p}^{2}=2m\int_{-\infty }^{+\infty }\rho_{x}Tdx-{\langle p\rangle }^{2}.$$

From (25), one obtains, for $T_{R}$:

(35) $$T_{R}=\frac{1}{2m}\left(P_{R}^{2}-P_{I}^{2}+\hbar \frac{\partial }{\partial x}P_{I}\right)=\frac{1}{2m}P_{R}+V_{qu,x}$$

with

(36) $$V_{qu,x}=-\frac{1}{2m}\left(P_{I}^{2}-\hbar \frac{\partial }{\partial x}P_{I}\right)=\frac{\hbar^{2}}{8m}{\left(\frac{\frac{\partial }{\partial x}\rho_{x}}{\rho_{x}}\right)}^{2}-\frac{\hbar^{2}}{4m}\frac{\partial }{\partial x}\left(\frac{\frac{\partial }{\partial x}\rho_{x}}{\rho_{x}}\right)=-\frac{\hbar^{2}}{2m}\frac{\frac{\partial^{2}}{\partial x^{2}}\sqrt{\rho_{x}}}{\sqrt{\rho_{x}}}$$

being the ”quantum potential” mentioned before. This contribution is entirely determined by the imaginary part $P_{I}$ of the complex hydrodynamic momentum and its derivative and, therefore, only depends on the amplitude of the wave function. Although the mean value of $P_{I}$ vanishes, the mean value of $V_{qu,x}$ does not.

The momentum uncertainty therefore contains two distinct contributions,

(37) $$\sigma_{p}^{2}=\left[\int_{-\infty }^{+\infty }\rho_{x}P_{R}^{2}dx-{\langle p\rangle }^{2}\right]+2m\int_{-\infty }^{+\infty }\rho_{x}V_{qu,x}dx=\sigma_{p,ph}^{2}+\sigma_{p,am}^{2},$$

i.e., one contribution from the phase and one from the amplitude.

The contribution from the phase depends on $P_{R}=\frac{\partial }{\partial x}S_{x}$ and vanishes if $S_{x}$ does not explicitly depend on the position; the contribution from the amplitude is always present.

In the momentum representation, a similar calculation leads to the position uncertainty $\sigma_{x}^{2}$, i.e.,

(38) $$\sigma_{x}^{2}=\langle \psi |{\hat{X}}^{2}|\psi \rangle -{\langle x\rangle }^{2}=\int_{-\infty }^{+\infty }\rho_{p}{\left(X^{2}\right)}_{M}dp-{\langle x\rangle }^{2}$$

where, again, the imaginary part of ${\left(X^{2}\right)}_{M}$ vanishes and the real part contains a contribution from the phase $S_{p}\left(p,t\right)$ and contributions from the amplitude $\sqrt{\rho_{p}\left(p,t\right)}$, according to

(39) $$\begin{array}{ccc} {\left(X^{2}\right)}_{R} & = & X_{R}^{2}-\left(X_{I}^{2}+\hbar \frac{\partial }{\partial p}X_{I}\right)\\ & = & -\left(\frac{\partial }{\partial p}S_{p}\right)-\frac{\hbar^{2}}{2}\left[\frac{\frac{\partial^{2}}{\partial p^{2}}\rho_{p}}{\rho_{p}}-\frac{1}{2}{\left(\frac{\frac{\partial }{\partial p}\rho_{p}}{\rho_{p}}\right)}^{2}\right]={\left(X_{R}\right)}^{2}-\hbar^{2}\frac{\frac{\partial^{2}}{\partial p^{2}}\sqrt{\rho_{p}}}{\sqrt{\rho_{p}}}. \end{array}$$

Defining a ”quantum potential” $V_{qu,p}$ in momentum space, in formal analogy with the one in position space (further details can be found in [28,29]), as

(40) $$V_{qu,p}=-\frac{m}{2}\omega^{2}\hbar^{2}\frac{\frac{\partial^{2}}{\partial p^{2}}\sqrt{\rho_{p}}}{\sqrt{\rho_{p}}},$$

(where, this time, the expression ”potential” is, physically, actually correct), the position uncertainty in momentum space (which should not be confused with the position uncertainty in position space; more details in Section 4) can then, again, be written as the sum of contributions from phase and amplitude as

(41) $$\sigma_{x}^{2}=\left[\int_{-\infty }^{+\infty }\rho_{p}X_{R}^{2}dp-{\langle x\rangle }^{2}\right]+\frac{1}{m\omega^{2}}\int_{-\infty }^{+\infty }\rho_{p}V_{qu,p}dp=\sigma_{x,ph}^{2}+\sigma_{x,am}^{2}.$$

Again, the contribution from the phase $X_{R}=-\frac{\partial }{\partial p}S_{p}$ vanishes if $S_{p}$ does not now explicitly depend on the momentum. Additionally, in this case, the contribution from the amplitude depends on the imaginary part of the complex quantity $X=X_{R}+iX_{I}$ and its derivative, providing a non-vanishing contribution, although $\langle X_{I}\rangle =0$.

This ”symmetry” between position and momentum representations is particularly important for stationary states where the phase (or at least its derivative) does not depend on the representation variable but is only explicitly time-dependent. In this case, the contributions from the phase to the uncertainties vanish; however, the ones from the amplitude do not, thus guaranteeing that Heisenberg’s principle can be fulfilled.

Finally, the correlation of position and momentum uncertainties, $\sigma_{xp}$, shall be given in the Madelung picture. This quantity can be written as (see, e.g., [30])

(42) $$\sigma_{x,p}=\frac{1}{2}\langle \hat{X}\hat{P}+\hat{P}\hat{X}\rangle -\langle x\rangle \langle p\rangle ,$$

which can be rewritten with the commutation relation (11) as

(43) $$\sigma_{x,p}=\frac{1}{2}\langle 2\hat{X}\hat{P}-i\hbar \rangle -\langle x\rangle \langle p\rangle .$$

In position representation, this leads with definitions (22) and (23) to

(44) $$\sigma_{x,p}=\int_{-\infty }^{+\infty }\rho_{x}xPdx-\langle x\rangle \langle p\rangle -i\frac{\hbar }{2}.$$

Taking into account the form of $P_{I}$ as given in (23), it is obvious that for square integrable wave functions,

(45) $$\int_{-\infty }^{+\infty }\rho_{x}xP_{I}dx=\frac{\hbar }{2}$$

is always fulfilled, guaranteeing that expression (44) stays real.

The correlation (44) can then be written as

(46) $$\sigma_{x,p}=\int_{-\infty }^{+\infty }\rho_{x}xP_{R}dx-\langle x\rangle \langle p\rangle =\int_{-\infty }^{+\infty }\rho_{x}\left(x-\langle x\rangle \right)\left(P_{R}-\langle p\rangle \right)dx.$$

It is important to note that although $\langle P_{R}\rangle =\langle \frac{\partial }{\partial x}S_{x}\rangle =\langle p\rangle$ is valid, the integral does not have to vanish if the phase $S_{x}$ explicitly depends on the position variable in a nonlinear way (e.g., quadratically, such as in the case of generalized coherent states, which is discussed in Section 4). The correlation, therefore, entirely depends on the phase of the wave function, and not at all on the amplitude. A similar calculation can also be performed in momentum space.

To give a more detailed illustration of the uncertainties and their correlations in position as well as in momentum space, in the next section, exact analytic solutions of the time-dependent Schrödinger equation, so-called generalized coherent states, i.e., Gaussian wave packets with time-dependent widths, will be considered.

## 4. Illustrative Example: Uncertainties and Their Correlations for Generalized Coherent States

### 4.1. Position Space

In this Section, our method of calculating uncertainties and correlations by means of amplitude and phase is illustrated using correlated coherent states, i.e., Gaussian wave packets with time-dependent maximums and time-dependent widths. The reason for this choice is that these states are the simplest analytical solutions of the time-dependent Schrödinger equation with non-trivial time-dependence of the uncertainties. For a complete analysis of the uncertainty relations with correlated coherent states, we refer to an outstanding paper by Dodonov et al. [31]. For more general potentials (at least in position space), the analytic form of *S* and ρ and the quantities depending on them will change, but the overall structure of our results and their dependence on *S* and ρ will not.

Gaussian wave packets with time-dependent maximums that obey the equations of classical mechanics and (in general) with time-dependent widths that are related to the quantum mechanical uncertainties are solutions of the time-dependent Schrödinger equation with a quadratic Hamiltonian. In position space, a general ansatz for such a wave packet (also called a generalized coherent state) can be written in the form

(47) $$\begin{array}{ccc} \langle x|\psi (t)\rangle & = & N_{x}\left(t\right)\exp\left[\frac{i}{\hbar }\left(\frac{m}{2}C{\tilde{x}}^{2}+\langle p\rangle \tilde{x}+K\left(t\right)\right)\right]\\ & = & N_{x}\left(t\right)\exp\left[-\frac{{\tilde{x}}^{2}}{2\sigma_{x}^{2}}+\frac{i}{\hbar }\left(\frac{m}{2}C_{R}{\tilde{x}}^{2}+\langle p\rangle \tilde{x}+K\left(t\right)\right)\right] \end{array}$$

with $\tilde{x}=x-\langle x\rangle$ and a complex time-dependent coefficient $C\left(t\right)=C_{R}+iC_{I}$ that fulfils the nonlinear Riccati equation $\dot{C}+C^{2}+\omega^{2}=0$. The purely time-dependent functions $N_{x}\left(t\right)$ and K(t) are not relevant for the following.

The imaginary part of C is related to the position uncertainty via

(48) $$C_{I}=\frac{\hbar }{2m\sigma_{x}^{2}}$$

with $\sigma_{x}^{2}=\langle x^{2}\rangle -{\langle x\rangle }^{2}=\langle {\tilde{x}}^{2}\rangle$. From the imaginary part of the Riccati equation,

(49) $$C_{R}=-\frac{1}{2}\frac{\frac{d}{dt}C_{I}}{C_{I}}=\frac{1}{2}\frac{\frac{d}{dt}\sigma_{x}^{2}}{\sigma_{x}^{2}}$$

follows (for details, see [32]).

The density $\rho_{x}\left(x,t\right)$, corresponding to the wave packet (47), can be written as

(50) $$\rho_{x}\left(x,t\right)={\left|N_{x}\left(t\right)\right|}^{2}\exp\left[-\frac{m}{\hbar }C_{I}{\tilde{x}}^{2}\right]=\sqrt{\frac{1}{2\pi \sigma_{x}^{2}}}\exp\left[-\frac{{\tilde{x}}^{2}}{2\sigma_{x}^{2}}\right].$$

Therefore, the position uncertainty is fixed by the width of the density $\rho_{x}$. This width, however, can be time-dependent, and the time-dependence enters the coefficient $C_{R}\left(t\right)$ in the phase of the wave function and contributes to the momentum uncertainty. Using our complex Madelung picture, this can be expressed as

(51) $$\sigma_{p}^{2}=\langle {\left(P^{2}\right)}_{R}\rangle -{\langle p\rangle }^{2}=\langle P_{R}^{2}-P_{I}^{2}+\hbar \frac{\partial }{\partial x}P_{I}\rangle -{\langle p\rangle }^{2}$$

with

(52) $$P_{R}=\frac{\partial }{\partial x}S_{x}=mC_{R}\tilde{x}+\langle p\rangle$$

and

(53) $$P_{I}=-\hbar \frac{\frac{\partial }{\partial x}\rho }{\rho }=\frac{\hbar }{2}\frac{\tilde{x}}{\sigma_{x}^{2}}.$$

According to (46), the position–momentum correlation $\sigma_{xp}$ can be written as

(54) $$\sigma_{xp}=\langle \tilde{x}P_{R}\rangle =mC_{R}\langle {\tilde{x}}^{2}\rangle =m\frac{d}{dt}\sigma_{x}^{2},$$

or, $C_{R}$ can be expressed in terms of the uncertainty correlation and the position uncertainty as

(55) $$C_{R}=\frac{1}{m}\frac{\sigma_{xp}}{\sigma_{x}^{2}}.$$

As has been shown in Section 3, the momentum uncertainty in position space has two contributions, one from the amplitude and one from the phase. In our case, these have the explicit form

(56) $$\sigma_{p,am}^{2}=-\left(\langle P_{I}^{2}\rangle -\langle \frac{\partial }{\partial x}P_{I}\rangle \right)=\frac{\hbar^{2}}{4}\frac{1}{\sigma_{x}^{2}}$$

and

(57) $$\sigma_{p,ph}^{2}=\langle P_{R}^{2}\rangle -{\langle P_{R}\rangle }^{2}=\frac{\sigma_{xp}^{2}}{\sigma_{x}^{2}}.$$

Therefore, the uncertainty product can be written as

(58) $$\sigma_{x}^{2}\sigma_{p}^{2}=\sigma_{x}^{2}\left(\sigma_{p,am}^{2}+\sigma_{p,ph}^{2}\right)=\frac{\hbar^{2}}{4}\left[1+{\left(\frac{2}{\hbar }\sigma_{xp}\right)}^{2}\right],$$

i.e., the minimum uncertainty $\frac{\hbar^{2}}{4}$ only depends on the amplitude of the wave function and is, therefore, also guaranteed for time-independent widths. If the uncertainty product is larger, this additional contribution originates entirely from the phase and is, in the case of our generalized coherent states, related to the time-dependence of the wave packet width that, again, reflects the correlation of position and momentum.

Thus, in position space, the uncertainties that determine the evolution of the wave packet are essentially the position uncertainty $\sigma_{x}^{2}$, which is responsible for the minimum uncertainty product and the position–momentum correlation $\sigma_{xp}$, and which provides additional contributions that depend, essentially, on the phase of the wave packet. The complex quantity C(t) that determines the time-evolution via a complex Riccati equation can be written using these contributions as

(59) $$C=\frac{1}{m}\frac{\sigma_{xp}}{\sigma_{x}^{2}}+i\frac{\hbar }{2m}\frac{1}{\sigma_{x}^{2}}.$$

### 4.2. Momentum Space

By analogy, in momentum space, a formulation is possible for the Gaussian wave packet that, in this case, can be formulated as

(60) $$\begin{array}{ccc} \langle p|\psi (t)\rangle & = & N_{p}\left(t\right)\exp\left[-\frac{i}{\hbar }\left(\frac{1}{2m}U{\tilde{p}}^{2}+\langle x\rangle \tilde{p}+L\left(t\right)\right)\right]\\ & = & N_{p}\left(t\right)\exp\left[-\frac{{\tilde{p}}^{2}}{2\sigma_{p}^{2}}-\frac{i}{\hbar }\left(\frac{1}{2m}U_{R}{\tilde{p}}^{2}+\langle x\rangle \tilde{p}+L\left(t\right)\right)\right] \end{array}$$

with $\tilde{p}=p-\langle p\rangle$ and the complex quantity $U=\frac{1}{C}$, where $U_{R}=\frac{C_{R}}{{\left|C\right|}^{2}}$, $U_{I}=-\frac{C_{I}}{{\left|C\right|}^{2}}$, and U fulfils the complex Riccati equation $-\dot{U}+\omega^{2}U^{2}+1=0$. Again, $N_{p}\left(t\right)$ and L(t) are not relevant for the following.

From the definition of U follows, for the imaginary part,

(61) $$U_{I}=-\frac{m\hbar }{2}\frac{1}{\sigma_{p}^{2}}$$

and, using (55), for the real part

(62) $$U_{R}=m\frac{\sigma_{xp}}{\sigma_{p}^{2}}.$$

The density $\rho_{p}\left(p,t\right)$ can be obtained in analogy with the one in position space. In this case, the momentum uncertainty is fixed by the width of $\rho_{p}\left(p,t\right)$, but now the time-dependence of the position uncertainty also has a contribution from the phase via $X_{R}$. Using the definition of the complex Madelung quantity as given in (27), the contribution to the position uncertainty from the amplitude is simply given by

(63) $$\sigma_{x,am}^{2}=-\left(\langle X_{I}^{2}\rangle +\langle \frac{\partial }{\partial p}X_{I}\rangle \right)=\frac{\hbar^{2}}{4}\frac{1}{\sigma_{p}^{2}}.$$

The contribution from the phase depends on $X_{R}=\frac{1}{m}U_{R}\tilde{p}+\langle x\rangle$ with ${\left(X^{2}\right)}_{R}=\frac{1}{m^{2}}U_{R}^{2}{\tilde{p}}^{2}+{\langle x\rangle }^{2}+\frac{2}{m}U_{R}\langle x\rangle \tilde{p}$ and $\langle X_{R}\rangle =\langle x\rangle$, and is given by

(64) $$\sigma_{x,ph}^{2}=\langle X_{R}^{2}\rangle -{\langle X_{R}\rangle }^{2}=\frac{1}{m^{2}}U_{R}^{2}\langle {\tilde{p}}^{2}\rangle =\frac{\sigma_{xp}^{2}}{\sigma_{x}^{2}}.$$

In this case, the uncertainty product takes the form

(65) $$\sigma_{p}^{2}\sigma_{x}^{2}=\sigma_{p}^{2}\left(\sigma_{x,am}^{2}+\sigma_{x,ph}^{2}\right)=\frac{\hbar^{2}}{4}\left[1+{\left(\frac{2}{\hbar }\sigma_{xp}\right)}^{2}\right],$$

i.e., the same as in position space.

The difference is that, now, in momentum space, the position uncertainty has two contributions, one from the amplitude and one from the phase, whereas, in position space, it was just vice versa. Like in position space, the minimum uncertainty entirely depends on the amplitude of the wave function. Additionally, in momentum space, the complex quantity that determines the time-evolution of the uncertainties via a Riccati equation can be entirely expressed in terms of the uncertainty in the chosen representation, here $\sigma_{p}^{2}$, and the position–momentum correlation $\sigma_{xp}$, i.e.,

(66) $$U=m\frac{\sigma_{xp}}{\sigma_{p}^{2}}-i\frac{m\hbar }{2}\frac{1}{\sigma_{p}^{2}}.$$

## 5. Conclusions

The analysis of the position and momentum uncertainties and their correlations in the framework of the (complex) Madelung picture shows that these quantities have an internal structure that is related to the complex structure of the quantum mechanical wave function. There is at least a formal similarity between the definition of our Madelung quantities and the weak values, but, as pointed out in Section 2.1, there are ontological differences involved that we do not intend to discuss in detail in this paper. However, we would like to refer interested readers to a recent paper by Pandey et al. [26], in particular, where an approach that is formally close to ours is discussed and also shows connections between weak values and modal theories such as Bohmian mechanics.

To avoid conceptual misunderstandings, the following points should also be clarified: The imaginary part of our complex momentum P in position space (see Equation (23)) has formal similarities with the so-called osmotic momentum used by Nelson [33,34] in his stochastic mechanics version of quantum mechanics. However, his quantity is purely **real** (with different signs for forward and backward derivatives in time), whereas our term is purely **imaginary**. This is an **essential** difference (even if the mean value of both terms vanishes).

There is also a complex version of Bohmian mechanics where the (real) action from the phase of the wave function is replaced by a complex action, following Schrödinger’s original introduction of the wave function in [4]. This leads to a complex momentum or velocity field, such as our momentum in Equation (23). However, from this, the incorrect conclusion is drawn that the independent (!) position variable must also be replaced by a complex one. Various attempts in this direction are in the literature, and all lead to a different physical situation than the one described by the original Schrödinger equation (see also our comment in [19], and the references cited therein).

In the context of our Madelung picture, momentum (37) and position (41) uncertainties, as well as their correlation (46), were calculated. It was shown that the momentum and position uncertainties, regardless of the potential considered, have a separable form, where the influence from the probability density ρ and the phase *S* of the projected quantum state can be distinguished. This was also illustrated particularly for the case of correlated coherent states or Gaussian wave packets (see Section 4). The uncertainties of the conjugate variable, however, have contributions from the amplitude as well as from the phase of the wave function. The contribution from the amplitude is the one that, in both cases, provides the minimum uncertainty $\frac{\hbar^{2}}{4}$ in the Heisenberg uncertainty product $\sigma_{x}^{2}\sigma_{p}^{2}$. The phase of the wave function can add contributions to this product, as long as it depends (more than linearly) on the variable of the representation. These contributions are usually (such as in the demonstrated case of Gaussian wave packets) time-dependent and can be expressed entirely in terms of the correlations of position and momentum uncertainties in both representations. It is well-known from the pioneering work by Dodonov et al [31] that, according to the Schrödinger–Robertson uncertainty relation [35,36], the difference between the minimum uncertainty product $\hbar^{2}/4$ and a larger value is due to the correlation of position and momentum. However, to our knowledge, it was never pointed out that this correlation term entirely depends on the phase of the wave function. This certainly has consequences for practical applications, such as the description of tunnelling phenomena. Does a description in terms of stationary states with a purely time-dependent phase suffice when the tunnelling dynamics actually explicitly involves contributions from the phase of the wave function? At least in the case of Gaussian wave packets that were analysed in more detail, an apparent symmetry between the description in both representations was found. Finally, it should be noted that the analysis of the uncertainties and correlations of Gaussian wave packets can also be performed in the Wigner formalism. This is, however, only complementary to our presentation here, as we have shown in [37] how the Wigner formulation is related to our Madelung picture. Therefore, any problem arising in one formulation could be tackled in the other. 

## Data Availability

Not applicable.
